# Sequence Types and Antimicrobial Resistance Profiles of *Streptococcus uberis* Isolated From Bovine Mastitis

**DOI:** 10.3389/fvets.2019.00234

**Published:** 2019-07-16

**Authors:** Nadine Käppeli, Marina Morach, Katrin Zurfluh, Sabrina Corti, Magdalena Nüesch-Inderbinen, Roger Stephan

**Affiliations:** Vetsuisse Faculty, Institute for Food Safety and Hygiene, University of Zurich, Zurich, Switzerland

**Keywords:** mastitis, *Streptococcus uberis*, sequence types, clonal complex 5, antimicrobial resistance

## Abstract

Bovine mastitis is one of the most common diseases among dairy cows and causes high economic losses in dairy industries worldwide. *Streptococcus uberis* is one of the most frequently identified pathogens causing the disease. In this study, 153 *S. uberis* strains isolated from mastitis milk samples were analyzed for their genetic diversity using multi locus sequence typing (MLST). Moreover, antibiotic susceptibility testing was performed using a microdilution assay and 11 antimicrobial agents including penicillin, which is the first line agent for treatment of bovine mastitis in Switzerland. MLST was successful for 152 (99.3%) of the strains. Overall, 103 different sequence types (STs) were determined, including 91 novel STs. *S. uberis* belonging to clonal complex (CC) 5 represented 47 (30.7%) of the mastitis cases. Two (1.3%) of the strains belonged to CC86 and one (0.7%) to CC143. The population structure identified in this work suggests that environmental transmission is the predominant route of infection in herds in Switzerland. Antimicrobial susceptibility testing determined a resistance rate of 11.8% for pirlimycin and elevated MIC90-values for marbofloxacin as well as for erythromycin. This study highlights the importance of genetic characterization of *S. uberis* and the need for veterinary breakpoints for surveillance of antimicrobial resistance in *S. uberis*.

## Introduction

Bovine mastitis is considered to be one of the most common diseases causing high economic losses in dairy industries worldwide (1). The economic losses include not only costs for treatment but deficits due to decreased quantity and quality of milk, higher number of culling and death of affected animals (2). Several studies reported *Streptococcus uberis* as one of the most frequently identified pathogens causing clinical cases of bovine mastitis (3–5). *S. uberis* is adapted to cattle and is classified as an environmental pathogen detected on various sites not only on the host but in bedding material, on milking utensils and other objects in the surroundings of cattle housing (6). Following the implementation of preventive control measures, the prevalence of clinical mastitis has declined in many countries during the last decades (7). Enhanced milking hygiene (correctly maintained equipment, teat disinfection, and personnel hygiene), antibiotic treatment and culling of persistently infected animals reduced the microbial load with contagious pathogens and therefore the risk for new infections. Nevertheless, the control of environmental pathogens like *S. uberis* presents an ongoing challenge for the management of dairy herds (8, 9). Despite the evidence supporting the essential role of *S. uberis* in the prevalence of bovine mastitis, only a limited amount of research has been carried out with regard to the characterization of *S. uberis* strains in Central Europe.

*Streptococcus* spp. are known to show good susceptibility to penicillin (9, 10) which is recommended by the Swiss society for veterinary science (SSV) as first line antimicrobial agent for intramammary treatment in cases of bovine mastitis caused by *Streptococcus* spp. and *Staphylococcus* spp. including *S. aureus* (11). The increasing numbers of resistant strains observed in association with the extensive use of antibiotics in human and veterinary medicine worldwide has led to the development of various monitoring programs in different countries. The possibility of resistant bacteria entering the food chain and the implicated risk to human health are only some of the reasons to enhance resistance surveillance. In the last two decades a growing number of publications have documented a slow but obvious decrease in beta-lactam susceptibility in streptococci (10). Even though most of the isolates examined in these studies were still considered susceptible to beta-lactam-antibiotics, this development underlines the need for further surveillance of antimicrobial resistance in order to ensure secure antibiotic treatment for future generations.

The aim of this study was to analyze the genetic diversity and the antimicrobial susceptibility profiles of *S. uberis* strains isolated from mastitis milk samples collected during a 1-year period from cattle on dairy farms in the area of the canton of Zürich, Switzerland.

## Materials and Methods

### Bacterial Isolation and Species Identification

The dairy farms selected for this study were customers of the ambulatory veterinary hospital of the University of Zürich that services the canton of Zürich and the surrounding region. The average herd size in the study region is 20–30 cows, and the most common dairy cow is the Swiss Brown. Clinical mastitis diagnosis was performed by the veterinarian in charge according to a standardized procedure. Milk samples were submitted to the routine mastitis diagnostic laboratory of the Institute for Food Safety and Hygiene in Zürich.

Milk samples were cultured according to standard procedures (12). Strains were identified using the Christie–Atkins–Munch-Petersen (CAMP) test as described by Munch-Petersen et al. (13), followed by an esculin hydrolysis (ESC) test. Briefly, a single colony was inoculated into an esculin broth medium (BioRad, Cressier, Switzerland) and incubated at 37°C for 24 h. After incubation, fluorescence was examined using an ultraviolet light source. Loss of fluorescence was interpreted as an ESC positive result. A *Streptococcus agalactiae* strain was used as an ESC negative control.

Species identification was confirmed by matrix-assisted laser desorption/ionization time of flight (MALDI-TOF) spectrometry (Bruker BioSpin AG, Fällanden, Switzerland) according to manufacturer's instructions. All *S. uberis* strains were cultured on sheep blood agar plates (24 h at 37°C), transferred into a glycerol freezing media and stored at −80°C until further processing.

Over a 1-year period (January 2017–January 2018), a total of 153 *S. uberis* strains were collected. Table S1 provides an overview of the strains used in this study.

### DNA Extraction

DNA isolation was performed after 24 h incubation in Brain-Heart-Infusion (BHI) medium at 37°C using the GenElute Bacterial DNA Kit supplied by SIGMA-ALDRICH (Darmstadt, Germany) following the manufacturer's instructions. A Nanodrop ND-1000 UV/Vis spectrophotometer (NanoDrop Technologies, Wilmington, DE) was used to determine the concentration of nucleic acids in the extractions.

### Multi Locus Sequence Typing (MLST)

MLST was performed using the GoTaq PCR system (Promega AG, Dübendorf, Switzerland) as well as primers and cycling conditions previously described by Coffey et al. (14). Briefly, target regions of *arcC, ddl, gki, recP, tdk, tpi*, and *yqiL* were amplified, PCR product purification as well as subsequent sequencing were outsourced (Microsynth, Balgach, Switzerland). Due to poor sequencing results a new forward primer was created for one of the seven housekeeping genes: *yqiL* fw new (5′-GGA TAC TCA TCT TGT GAG ACG ATT AAA GG-3′). Reverse primer and cycling conditions remained unchanged. Sequence data analysis was performed using CLC Main Workbench 8.0.1. The allelic profile of each strain was identified and, where possible, strains were assigned to sequence types (ST) using the PubMLST sequence and profile definitions database (https://pubmlst.org). Currently unidentified alleles of each housekeeping gene as well as unknown allelic profiles were submitted to the database and new sequence types (ST) were generated. Data was visualized using the platform independent JAVA software phyloviz 2.0 and the goeBURST algorithm (15).

### Antimicrobial Susceptibility Testing

Minimum inhibitory concentrations (MIC) of various antimicrobial agents were determined by the microdilution assay using the MICRONAUT-S Mastitis 3 system (MERLIN, Bornheim-Hersel, Germany) according to manufacturer's instructions. The test system included the following antimicrobial agents: penicillin, ampicillin, cefazolin, cefoperazone, cefquinome, oxacillin, pirlimycin, erythromycin, marbofloxacin, amoxicillin/clavulanic acid, and kanamycin/cephalexin,

The MIC_50_ and MIC_90_ values (MICs at which at least 50 and 90% of the isolates in a test population are inhibited, respectively) were determined.

## Results

In total, 153 *S. uberis* strains were isolated from milk samples originating from 54 herds, with the majority (87%) of the farms located in the canton of Zurich. Twenty-six (48%) of the 54 herds were sampled more than once with every sample deriving from a new case of mastitis diagnosed by the veterinarian in charge. Acute mastitis was diagnosed in 68 cases, subclinical in 17, and chronic mastitis in 14 cases, respectively. For 54 cases, the type of mastitis was unspecified (Table S1).

MLST identified 96 different alleles among the seven gene fragments, whereof 21 were found for the first time in this study. Between one and six new alleles per housekeeping gene were detected among the 153 *S. uberis* isolates. The highest number of new alleles was detected for *tdk* with six new alleles, and *ydiL* with five new alleles, respectively (Table S1). The nucleotide sequences of the novel alleles are registered in the *S. uberis* MLST sequence and profile definitions database (https://pubmlst.org) and can be accessed for each locus using the allele IDs listed in Table S1.

Based on the allelic profiles, 152 strains were assigned to 103 different STs, whereof 91 (88.3%) were newly described STs. One isolate (SU162) did not generate an amplification product for the locus *yqiL* and was therefore not assigned an ST.

The most frequently detected ST was ST896 (*n* = 10). This ST was novel and belonged to clonal complex (CC)5, together with further 22 novel STs (*n* = 29) and previously described ST6, ST22, ST316, ST241, and ST361 (*n* = 8). In total, CC5 contained 47 strains from 30 farms (Table S1).

**Figure 1:**
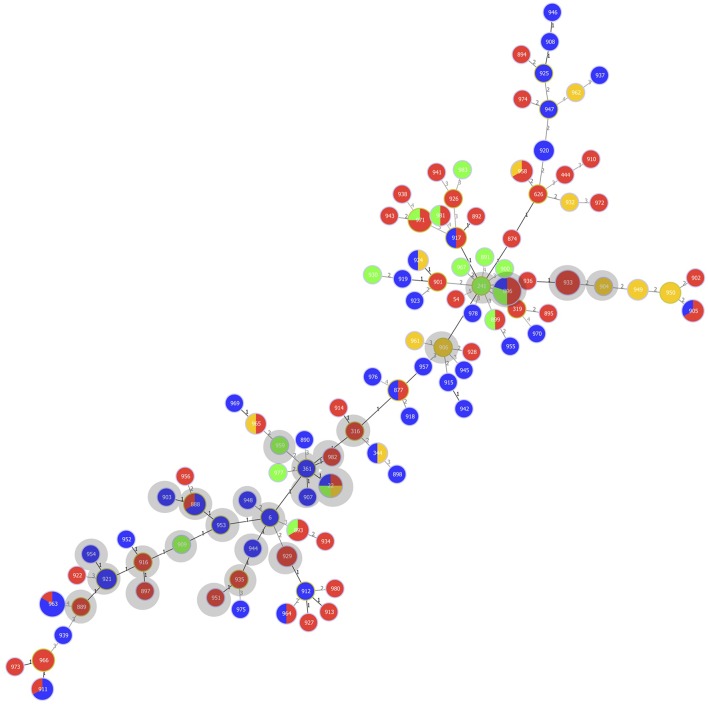
Multilocus sequence type (MLST)-based minimal spanning tree of 152 *Streptococcus uberis* isolated from bovine mastitis milk during 2017 in Switzerland. The tree was calculated and generated using the goeBURST full MST algorithm in Phyloviz 2.0. Node sizes reflect the number of isolates with specific MLST profile. Numbers within the nodes indicate the ST. Node colors refer to types of mastitis: acute (red), subclinical (green), chronic (orange), unspecified mastitis (blue). Numbers on lines indicate locus variants between adjacent nodes. Grey shadows indicate STs belonging to clonal complex (CC)5.

The goeBURST analysis indicated three possible founder STs within CC5 (ST6, ST241, and ST361). ST6 contained one isolate and had one isolate that was a single locus variant (SLV) and five strains that were double locus variants (DLVs). ST241 consisted of one isolate and had one isolate that was an SLV and 15 strains that represented DLVs. ST361 (one strain) had six strains that were SLVs strains and one strain representing a DLV (Figure 1).

Of the 47 strains belonging to CC5, 22 (46.8%) were associated with acute mastitis, 7 (14.9%) with subclinical, and 3 (6.4%) with chronic mastitis. For the remaining 15 (31.9%) of the strains, the type of mastitis was unspecified (Figure 1 and Table S1).

Further clonal complexes included CC86 (*n* = 2), and CC143 (*n* = 1), whereof both ST891 and ST962 (CC86) were novel (Table S1).

The remaining 73 STs (*n* = 102) did not belong to any CC and consisted of 67 novel STs (*n* = 94) and six previously described STs (*n* = 8). Thereof, 45 (44.1%) were associated with acute mastitis, 9 (8.8%) with subclinical, and 10 (9.8%) with chronic mastitis. For 38 (37.3%), the type of mastitis was unspecified (Figure 1 and Table S1).

**Figure 2:**
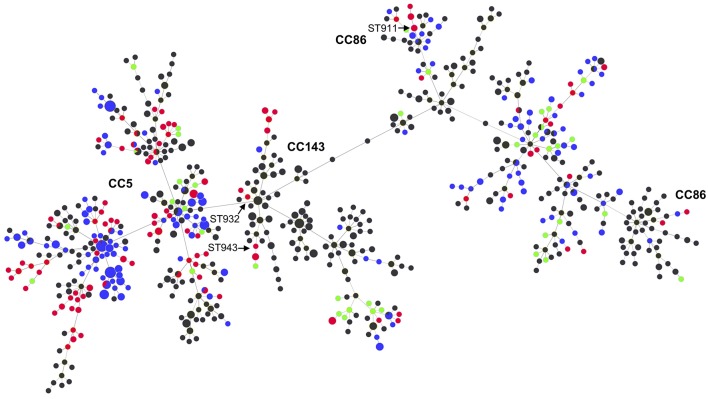
MLST-based minimal spanning tree of all *S. uberis* isolates in the *S. uberis* MLST database (pubmlst.org/suberis). Node colors refer to origin of isolates: this study (red), Switzerland during 2008 (green), UK (blue), other countries (dark gray). Node sizes reflect the number of isolates with specific ST. Clonal complexes are indicated in bold. STs from this study that are discussed in the main text are indicated with arrows.

Figure 2 shows the relationship between the STs of this study and those reported globally in the *S. uberis* MLST database (pubmlst.org/suberis).

**Figure 3:**
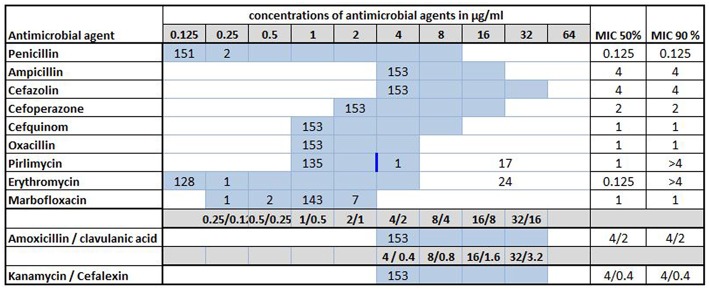
Minimal inhibitory concentrations (MICs) of 11 antimicrobial agents for 153 *Streptococcus uberis* isolated from bovine mastitis milk during 2017 in Switzerland. Numbers indicate the number of strains exhibiting the corresponding MIC value. Light blue areas represent the tested concentration range on the microdilution plate. Values above this range denote MIC values greater than the highest concentration tested. The breakpoint (≥4 μg/ml) for pirlimycin is indicated by a blue vertical bar. MIC_50_ and MIC_90_ values represent the concentrations of antimicrobial agents inhibiting growth of 50 or 90% of the strains, respectively.

Antimicrobial susceptibility testing was performed for all 153 *S. uberis* strains investigated in this study. A summary of the MICs and the MIC_50_/MIC_90_ values is presented in Figure 3. Of the antimicrobial agents tested, pirlimycin is the only one with a defined breakpoint (≥4 μg/ml) for *Streptococcus* spp. isolated from cattle mastitis (16). Thereby, 18 (11.8%) strains originating from nine different farms (farms F8, F15, F16, F22, F30, F31, F35, F36, and F51, respectively) were resistant to pirlimycin while MICs remained ≤ 1 μg/ml for the remaining 135 (88.2%) of the isolates, which was the lowest measurable concentration for this agent in the MICRONAUT assay (Table S1). A total of 24 strains (15.7%) from 13 farms were able to grow at all tested dilutions of erythromycin in the assay, setting the MIC_90_ at >4 μg/ml (Figure 3). Seventeen of these strains were among those resistant to pirlimycin, as mentioned above. The remaining seven originated from farms F1, F4, F13, F25, F31, and F43, respectively (Table S1).

For seven (4.6%) of the strains the MICs of marbofloxacin were 2-fold higher (≥2 μg/ml) than the MIC50 and MIC90 (both 1 μg/ml, respectively). For two (1.3%) of the isolates, the MICs of penicillin were slightly increased (0.25 μg/ml), but remained ≤ 0.125 μg/ml for the remaining 151 strains.

All 153 isolates tested in this study showed a good susceptibility to ampicillin, cefazolin, cefoperazone, cefquinom, oxacillin, and kanamycin-cephalexin, with MICs of 4, 4, 2, 1, 1, and 4/0.4 μg/ml, respectively (Figure 3). All values represent the lowest measurable concentrations in the MICRONAUT assay. All isolates also exhibited good susceptibility to amoxicillin/clavulanic acid, with MICs of 4/2 μg/ml).

## Discussion

In this study, we assessed the genetic relatedness and the antimicrobial resistance of *S. uberis* causing bovine mastitis on 54 farms in Switzerland during 2017. The main limitation of this study was the restriction to one geographical area in Switzerland. However, farm type, breed of dairy cows and herd size were typical for the Swiss Midlands. The analysis performed revealed that the 153 isolates belonged to 103 STs, with 30.7% of the isolates assigned to CC5. This CC has been identified as the major lineage among *S. uberis* isolates causing bovine mastitis in the UK (14, 17). Our data confirm the wide distribution of this lineage. Other previously described *S. uberis* CC86 and CC143 were detected at very low rates of 1.3 and 0.7%, respectively, supporting earlier reports that these clones are rare in Europe compared to Australia and India (14, 17–20).

Within CC5 we identified a high number of STs, including STs described for the first time in this study. The high diversity of STs both within CC5 as also among the other STs, suggests a heterogeneous, rather than a contagious dissemination of *S. uberi*s among the dairy cattle from the farms analyzed in this study, e.g., through environmental contamination. The goeBURST analysis showed that the Swiss strains from 2017 were distributed among CC5, CC86, CC143 in the global population of *S. uberis* (Figure 2). There were some inconsistencies where one isolate belonging to ST932, which is an SLV of ST184 (CC143), and ST943, which is a DLV of ST124 (CC143), were not assigned to this CC by the MLST database (Figure 2). Likewise, ST911, an SLV of ST22 (CC86) was not assigned to CC86. These observations indicate that these CCs may be more prevalent than suggested by the database.

The comparison of the STs from this study with those of 38 *S. uberis* strains submitted to the isolate database at pubmlst.org/suberis from Switzerland during 2008 shows only one common ST (ST319) (Figure 2). This is a further indication that the majority of *S. uberis* from Swiss dairy cattle represent unique genomic backgrounds and lack predominant contagious STs such as ST5, ST6, ST20, and other STs of the CC5 compared to other European countries such as the UK (17).

Nevertheless, of the 54 herds examined, 13 (24%) had two or more individual cows infected with *S. uberis* that belonged to identical STs. For one herd (farm 14), five cases of acute mastitis were associated with ST933 (CC5), suggesting contagious events. Furthermore, for three herds, four cases each of either acute mastitis or unspecified mastitis associated with ST896 (CC5) on farm 16, ST963 on farm 31, and ST971 on farm 14 were observed. While these data suggest potential transmission events, this study is limited by the lack of detailed recordings of the clinical cases within the affected herds. More precise documentation on the temporal patterns of the occurrence of specific STs is needed in order to identify potentially contagious transmission episodes. Moreover, further molecular mechanisms that may promote the higher prevalence of specific STs such as virulence factors, were not analyzed in this study. Additional investigations are required to characterize such factors in various *S. uberis* STs, including factors affecting biofilm formation (21), host cell invasion, survival in the host environment or evasion of host immune response, respectively (18, 22, 23).

In the present study, we determined MICs of 11 antimicrobial agents, including penicillin, which is the first line antimicrobial according to the prescription policy of the ambulatory veterinary hospital of the University of Zürich, and amoxicillin/clavulanic acid which is the second-line drug of choice for the treatment of *S. uberis* mastitis. Erythromycin was tested as a representative of macrolides, which are third-line antimicrobials recommended by the SGV for the treatment of *S. uberis* infections (11). The other agents were tested for epidemiological purposes.

Due to the lack of interpretive criteria for mastitis strains, an accurate classification into susceptible or resistant strains, as well as the specification of a resistance rate was possible for the lincosamide pirlimycin only. Pirlimycin resistance was limited to nine farms and within these farms, five had two or more individual cows infected with resistant isolates. Notably, on farm eight and farm 31, resistant isolates were associated with ST963, which is potentially a contagious ST, as mentioned above. The majority of the pirlimycin resistant isolates in this study also exhibited erythromycin MICs of >4 μg/ml. *S. uberis* with this resistance phenotype harboring *ermB* have been described from bovine mastitis cases in France (24). Additional studies are needed to determine the resistance genotype of the isolates described in this study. Furthermore, monitoring the development of resistance in *S. uberis* is required.

In the absence of veterinary clinical breakpoints, a potential alternative to monitor the development of antimicrobial susceptibility of animal pathogens like *S. uberis* is to track an increase or decrease of MIC_50_/MIC_90_ values over time. Reports providing such data for Switzerland are scarce. A study analyzing 208 *S. uberis* isolates in 2013 recorded MIC_50_/MIC_90_ values for penicillin of ≤ 0.12 μg/ml/ ≤ 0.12 μg/ml, similar to our data (25). However, a direct comparison is not feasible due to differences in laboratory methods. Only one recently published study presenting the results of an ongoing long-term susceptibility monitoring program in European countries (VETPATH) includes MIC_50/_MIC_90_ values (26). Compared to our study, the *S. uberis* strain collection examined in the VETPATH program expressed higher MIC_90_ values for penicillin and marbofloxacin with 0.25 and 2 μg/ml, respectively, while the values for the combination of kanamycin-cephalexin were identical with those determined in our study. Due to a strong variance in the dilution range tested, the differences in the MIC_50_/MIC_90_-values for amoxicillin-clavulanic acid, cefquinome and erythromycin could not be compared between the two studies.

Ignoring potential interspecies differences in pharmacokinetics as well as the fact that *S. uberis* is a cattle-adapted species, we attempted to determine and compare resistance rates using breakpoints published for human viridans streptococci (16), as described previously for the pan-European monitoring program VETPATH (21). There, resistance rates of 0.0% and 20.2% for penicillin and erythromycin, respectively, were calculated for *S. uberis*. Using the same breakpoints (≥4 μg/ml for penicillin and ≥1 μg/ml for erythromycin), similar resistance rates of 0.0% for penicillin and 15.7% for erythromycin, respectively, were observed for our strain collection. Furthermore, we observed a resistance rate of 0.0% for ampicillin (breakpoint ≥ 8 μg/ml), an agent that was not included in the VETPATH study. In contrast, the collected data from the French surveillance program (RESAPATH), was interpreted using different breakpoints published by the veterinary section of the Comité de l'antibiogramme de la Société Française de Microbiologie (CA-SFM) for *Streptococci* spp. (27, 28). Unsurprisingly, the French study presented resistance rates for *S. uberis* similar to those of the pan-European study with 0.0% for penicillin and 20.0% for erythromycin. Moreover, using the CA-SFM breakpoint for ampicillin (16 μg/ml) classified the resistance rate of resistance at 0.0%, accordingly.

With the aim of providing interpretive criteria and of harmonizing diagnostic procedures for mastitis isolates, Fessler et al. (29), determined the MICs as well as disc diffusion diameters of a total of 1,086 bovine clinical isolates challenged with cefoperazone. Considering pharmaceutical studies and treatment recommendations, they proposed to classify the different species tested, including *S. uberis*, as susceptible to cefoperazone when the MIC is ≤ 2 μg/ml, intermediate when the MIC is 4 μg/ml and resistant when the MIC is ≥8 μg/ml. Utilizing these breakpoints for interpretation of our data, we considered 100% of our isolates as fully susceptible to cefoperazone.

Our study underlines the need for veterinary clinical breakpoints in order to improve surveillance data and optimize treatment of bovine mastitis and other animal diseases.

## Author Contributions

RS and SC designed the study. NK, MM, KZ, and SC carried out the microbiological and molecular biological tests. NK, RS, and MN-I analyzed and interpreted the data. NK and MN-I drafted the manuscript. All authors read and approved the final manuscript.

### Conflict of Interest Statement

The authors declare that the research was conducted in the absence of any commercial or financial relationships that could be construed as a potential conflict of interest.
